# Evaluation of the Obesity Prevention, Blood Glucose, and Blood Lipid Control of Vietnamese Rice Varieties in High-Fat Diet-Induced Obese Mice

**DOI:** 10.1155/2021/4880603

**Published:** 2021-09-07

**Authors:** Thuy Linh Nguyen, Khanh Son Trinh

**Affiliations:** ^1^Nong Lam University, Ho Chi Minh City, Vietnam; ^2^Ho Chi Minh City University of Technology and Education, Ho Chi Minh City, Vietnam

## Abstract

Five Vietnamese rice varieties, which differ in their basic chemical composition (starch content, amylose content, fiber content) including polished rices and whole grain rices, were chosen for this study. High-fat diet-induced obesity, using these rice, was subjected to in vivo experiments to evaluate the effects of body weight gain, caloric intake, postprandial blood glucose level (PBGL), and glycemic index (GI) in tested mice groups. PBGL peaks appeared at 30^th^ minute after eating, and GI of each experimental group was ranked in order as GN > TL > HR > GM > LĐ and GN > LT > HR > LĐ > GM, respectively, in which, brown rice (LĐ) and germinated brown rice (GM) had low GI. Furthermore, these rice varieties caused the lowest LDL cholesterol and the ratio of LDL/HDL cholesterols in mice. In this study, the higher the amylose and fiber contents were, the lower glycemic index, triglycerides, LDL, and LDL/HDL values were. The golden flower glutinous rice (GN), with low amylose and fiber content, gave worse blood lipid parameters than that of GM and LĐ. Histopathological studies of white adipose and liver tissues showed that LĐ and GM significantly reduced the symptoms of obesity and fatty liver compared with the others, especially compared to GN. The results obtained from this study help patients with overweight, obesity, and type 2 diabetes choose the right rice variety for their daily diet to be able to control their diseases.

## 1. Introduction

Base on the statements of WHO [1], overweight and obesity are the excess of fat accumulation and can have adverse health effects. Between 1975 and 2016, worldwide obesity rates tripled. In 2019, estimate more than thirty-eight million children under the age of five is overweight and obese. About fifty percent of under five-year age children with overweight and obesity lives in Asia. Not only appears in high-income country, overweight and obesity are on the rise in low- and middle-income countries. The main reason of overweight and obesity is an inequality between consumed and expended calories. Being overweight and obesity can be the main reasons of diseases such as cardiovascular diseases, diabetes, musculoskeletal medical (especially osteoarthritis), and some cancers. However, overweight, obesity, and their related no communicable diseases can be avoided following below recommendations: (a) limit calories intake from total fat and sugar; (b) increase consumption of whole grain, whole nut, fruit, and vegetable; and (c) engage in regular physical activity [1].

Rice (*O. sativa* L.), especially polished white rice, is the most widely consumed food and the major source of carbohydrate for the majority of the world's population. Rice provides around 35-59% daily caloric intake for the human body [2, 3]. White rice is polished from brown rice. As a result, white rice lost its fiber, vitamins, polyphenols, crude fat, and protein. The use of low-GI brown rice, instead of white rice, may minimize the risk of obesity [4]. Adequate diet is the foundation for the elimination and control of diabetes, overweight, and obesity due to its primary concentration at maintaining a low and stable postprandial blood glucose level. Low-GI carbohydrates may reduce the risk of type 2 diabetes mellitus and cardiovascular disease [2].

Compared to amylopectin-rich meal, a previous study that reported rich amylose meal causes lower glucose peak [5]. Rich amylose diet may be of potential to diabetic patients. Whole grain rice contains high nutritional and biological substances such as fiber, minerals, vitamins, and *γ*-oryzanol. Many previous studies showed evidences that these components can reduce low-density lipoprotein level, total cholesterol level, and control blood pressure and can prevent colorectal cancer [6–8].

This study compared the effects of five Vietnamese rice varieties (including various amylose/amylopectin ratio and fiber contents) in high-fat diet-induced obese mice. The differences in body weight gain, postprandial blood glucose level, blood lipid, histopathology of liver, and adipose tissue were investigated.

## 2. Materials and Methods

### 2.1. Animals and Husbandry

Experimental animals were male mice (*M. musculus* var. *albino*) provided by Pasteur Institute of Ho Chi Minh City, weighing from 16-20 g/individual. Mice were raised at room temperature (about 30°C, 50% relative humidity) with a controlled 12 h dark/light cycle and were allowed to drink freely. Mice were acclimatized for a week with an AIN-93 M diet [9]. Diets and distilled water were provided ad libitum to mice all through the experiments.

After 1 week of adaptation, during next five weeks, tested mice were divided to five groups. These groups were fed different high-fat diets (HFD) (Table 1) with various rice flours. Tested individual was fed 2.0 g of meal/individual/day.

Experimental procedures were obtained ethical clearance with reference No. IRB-A-2020 (Institutional Review Board at Dinh Tien Hoang Institute of Medicine has the operating code as IRB-VN02010 issued by Vietnam Ministry of Health on 15th October 2015). Sample size (six individuals per group) was calculated following a previous study [10].

### 2.2. Chemical Compositions of Rice Flours

Content of moisture, ash, crude fat, crude protein, and crude fiber in flour were, respectively, analyzed following AOAC 925.10, AOAC 923.03, AOAC 920.39, AOAC 984.13, and ISO 5498 : 1981 [11, 12]. Total carbohydrate and starch (available carbohydrate) were calculated following FAO [13].

### 2.3. Glucose Tolerance Test

After 1-week adaptation, mice were conducted by the glucose tolerance test [14]. This test determined the postprandial glucose level in the plasma after meal. Mice were divided into five groups (6 individuals/group) according to their HFD diet (Table 1). Mice were fasted for 16 h and then fed glucose (0.5 ml, 7.5%) or autoclaved rice flour (0.5 ml, 7.5%, w/v) via an oral Zonde needle. After eating, a drop of blood was taken from the tail vein of mouse at 0, 30, 60, 90, 120, 150, 180, and 240 min [15, 16]. Blood glucose level was measured with an Accu-Chek Active Glucose System (Roche Ltd., Basel, Switzerland).

### 2.4. Blood Biochemistry and Histopathological Examination

A volume (around 1.0 ml) of blood was collected using the cardiac puncture technique [16–18]. Levels of total cholesterol (TC), triglycerides (TG), high-density lipoprotein cholesterol (HDL), and low-density lipoprotein cholesterol (LDL) were measured by Beckman Coulter AU480 and Diasorin Liaison XL instruments.

Liver and white adipose tissues were collected, fixed in 10% formalin solution and embedded in paraffin. A four *μ*m-thick serial section was prepared and stained with hematoxylin and eosin (H&E) for light microscopic examination [8].

### 2.5. Changes in Body Weight

Changes in body weight of tested groups were measured every week. Before measurement, mouse was fasted overnight (without water feeding) [19]. The difference of mouse's body weight was calculated as its body weight gain (BWG).

### 2.6. Amylose Content

The amylose content was determined following a previous method of Zhu et al. [20]. The absorbance (Abs) of a solution of amylose-iodine complex at 620 nm and 510 nm was recorded.

(1) $$\begin{array}{c} \text{Amylose }\left(\%\right)=\frac{\text{Abs}620–\text{Abs}510+0,0542}{0,3995}. \end{array}$$

### 2.7. Glycemic Index and Caloric Intake

The glycemic index (GI) was estimated by using the incremental area under the glycemic response curve of food relative to a similar amount of oral glucose: GI = (*S*_*R*_/*S*_glucose_) × 100, where *S*_*R*_ was the area under a curve of blood glucose level from 0.5 ml autoclaved rice flour (7.5%, w/v), and *S*_glucose_ was the area under a curve of blood glucose level from 0.5 ml of glucose (7.5%, w/v) (Merck, Germany) [21]. The Origin 8.5.1 software (OriginLab Corporation, Northampton, USA) was used to calculate above areas.

Caloric intake of mice was calculated following an equation: *E* = GI × *E*_glucose_, where *E*_glucose_ = 4 × *G* (kcal), 4 is the Atwater index (kcal/g), indicating that 1.0 g of glucose releases 4 kcal after digestion, and *G* (g) is the mass of glucose feed [22].

### 2.8. Statistical Analysis

All experiments were carried out in triplicate and shown as mean ± standard deviation. Statistical analyzes, including ANOVA and Duncan test (*p* < 0.05), were conducted with SPSS statistical program package (version 20.0, USA).

## 3. Results and Discussions

### 3.1. Chemical Composition of Rice Varieties

Table 2 shows the basic chemical components of rice flours. Compared to the others, golden flower glutinous rice (GN) and red rice (HR) had the highest total carbohydrate and starch content, but GN had the lowest amylose content [23]. Actually, amylose and crude fiber contents of samples were orderly arranged following: GN < TL < HR < LĐ < GM. Meanwhile, germ rice (GM) had the lowest carbohydrate and starch content. The remaining components of rice flours were not so much different. Studies showed that amylose-rich foods reduce blood glucose levels at 30^th^ minutes after eating compared with that of amylopectin-rich foods [5]. The process of milling to remove bran husks (jasmine rice, yellow flower sticky rice) resulted in the loss of some substances of high nutritional value such as fiber, minerals, vitamins, and *γ*-oryzanol [24]. These substances are beneficial for the human health because they help stabilize blood glucose and reduce cholesterol in the blood [7, 25].

### 3.2. Body Weight Gain (BWG)

**Figure 1:**
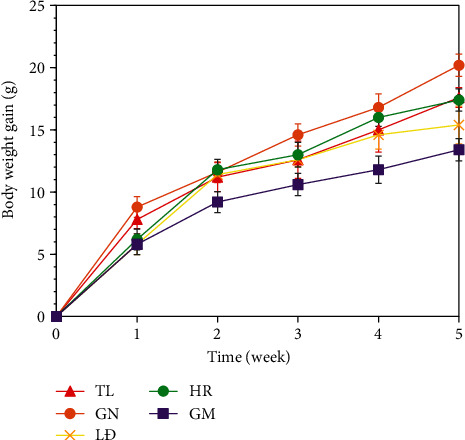
Body weight gain of mice.

The body weight of HFD mice increased rapidly during five weeks of experiment. There were significant differences in body weight gain (BWG) between groups (*p* < 0.05) (Figure 1). The BWG values of the HFD mice were sorted in the order: GN > TL > HR > LĐ > GM. Practically, the trend of BWG was almost opposite to that of amylose and fiber contents. In other words, the higher the amylose and fiber contents were, the lower the body weight gain of mice was. In this study, GM and LĐ had the highest amylose content (20.68 and 21.63%, w/w) and highest fiber content (1.60 and 1.14%, w/w). Some previous studies showed the effect of amylose on starch digestibility. Zhu et al. [26] demonstrated the high amylose content results in the high-resistant starch contents. Previous studies also showed that dietary fiber and amylose-rich foods could lower blood glucose level after consumption, which in turn helps control the overweight/obesity and further type 2 diabetes mellitus [27]. Evidently, fiber helped to interfere with starch absorption and helped to stabilize blood glucose level. In addition, some substances with high biological activity in rice bran such as phenolic, *γ*-oryzanol, vitamin E, and carotenoid helped to stabilize blood glucose level [6]. In particularly, gamma-aminobutyric acid (GABA) in sprouted rice had health benefits by lowering blood pressure [8].

### 3.3. Postprandial Blood Glucose Level, Caloric Intake, and Glycemic Index

**Figure 2:**
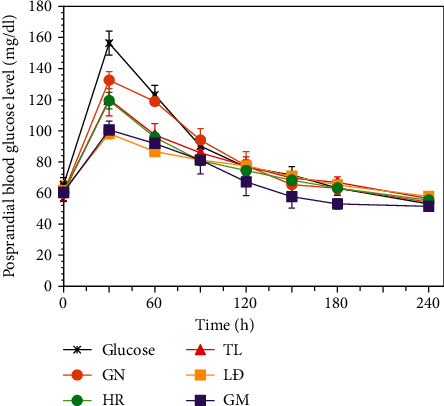
Postprandial blood glucose tolerance in mice.

Figure 2 shows the postprandial blood glucose level (PBGL, mg/dl) curves of the tested groups of mice. Fasting (basal) blood glucose level (FBGL) of mice was around 60 mg/dl. In all groups of mice, after eating, blood glucose levels increased rapidly, reached their maximum after 30 minutes, and then decreased gradually for all diets. The change of PBGL in the control group (using 0.5 ml of 7.5% glucose solution) was similar to the previous studies [28, 29]. After 30 minutes, PBGL_max_ of mice, which using rice flours, was arranged in an order: glucose > GN > TL > HR > GM > LĐ. After 240 minutes, PBGL of all experimental groups gradually lowered to FBGL level.

It can be seen that the BWG, caloric intake, and GI values of the experimental groups of mice were in opposite relations. Our study has shown the lower potential for body weight gain of GM, LĐ, and HR in HFD diets compared to that of GN and TL. This has also been found in many previous studies [8, 30]. Theoretically, the glycemic index (GI) is used to assess the increase in plasma glucose levels after eating a certain amount of carbohydrate in a test food, compared to the similar quantity of carbohydrate from a control food (white bread or glucose solution). These measurements were done the same of not only tested individuals but also conditions of experiment. The calculations use the initial blood glucose concentrations as a baseline [31]. Caloric intake was the energy that each mouse gets from its diet.  Table 3 shows the caloric intake and glycemic index values of the tested mice groups in an order: glucose > GN > TL > HR > LĐ > GM. Interestingly, in this study, there were many interdependences between the chemical compositions (amylose and fiber contents) of rice varieties and the physiological values (caloric intake, GI, BWG) of test mice. According to postprandial glycemic index of foods, GI is stated as (i) low (≤55), (ii) medium (56-69), and (iii) high (≥70) [27]. Thus, GM and LĐ were low GI rice, HR was medium GI rice, and TL and GN were high GI rice. Ludwig et al. [32] showed that a low-GI meal would limit the rapid increase of postprandial glucose level in blood and reduces a sequence of hormones and metabolic changes. Besides, low-GI diet reduces hunger. All of these factors lead to reducing of food intake. Low-GI meal may provide many physiologic benefits in serum lipids and reduce risk of diabetes mellitus and other diseases associated with hyperinsulinemia [32].

### 3.4. Blood Lipid Parameters

Table 4 shows the plasma lipid profiles in mice. At the beginning of the experiment, the blood lipid values of the tested groups did not differ significantly (*p* < 0.05). Values (mg/dl) of TC, TG, and HDL were <45, <100, and 50.8 ± 3.3, respectively. After five weeks, mice, which were fed on GN diet, exhibited significant elevation of TC, TG, LDL, and TC/HDL compared to that of the others. However, the feeding of GM, HR, and LĐ diets resulted significant reductions of above parameters and higher HDL level compared with the others. Especially, TC, TG, LDL, and TC/HDL-cholesterol levels of the GM group were the lowest compared to that of the other rice varieties. Obviously, in this study, the higher the amylose and fiber contents were, the lower glycemic index, triglycerides, LDL, and LDL/HDL values were.

Cholesterol is the biological lipids found in humans and animals. Cholesterol is transported in the blood circulation in form of cholesterol-containing lipoprotein particles (LDL and/or HDL). About 30% of body cholesterol comes from dietary sources. If body cholesterol to much or too little, the risk of several diseases increases. Low LDL can increase risk of abnormalities such as cancer, premature birth, anxiety, and depression. However, high LDL may increase the risk of coronary artery disease and stroke. Several previous studies had demonstrated an association between elevated levels of TC and LDL, the reduced levels of HDL, and coronary artery disease. Besides, the ratio of TC/HDL and LDL/HDL was suggested for risk assessment and disease prognosis. Many studies show that whole grains, rich in fiber, effectively lower blood cholesterol [33].

Zavaroni et al. [34] showed that high blood glucose level caused high TC and TG levels in the blood, thereby increasing the risk of coronary diseases and noninsulin-dependent (type 2) diabetes. In particular, a low-GI diet reduces the amount of TC and TG in blood [35]. Furthermore, the use of amylose-rich starch is beneficial for obese and diabetic patients by helping to reduce the amount of TC and TG in blood. Denardin et al. [36] stated a diet rich in amylose resulted in a decrease in serum triglycerides compared to a diet rich in amylopectin. In our study (Tables 2 and 4), a diet rich in amylose also caused the lower TC value. Specifically, the GN and GM diets, containing the lowest (2.86%) and the highest (20.68%) amylose content, showed the low TC values in mice of 129.8 and 80.0 mg/dl, respectively, compared to the others.

These results above were explained by the relationship between starch digestibility and its effect on glucose hepatic metabolism. During digestion, starch was hydrolyzed to glucose and is delivered to the liver in three main ways: “(a) to maintain its concentration sufficiently high to supply energy to the brain and other tissues; (b) converted into glycogen, stored in the liver and muscles; (c) converted into fatty acids, transported by the triglycerides” [37]. Compared to amylose, the amylopectin molecules are more easily digested; so, more glucose will be transferred to the liver. These glucose molecules are rapidly converted into fatty acids, transported by triglycerides (TG) and cholesterols (TC), and then stored in the adipose tissue. These increase the TC and TG in the serum. Thus, a rich amylose diet can be beneficial for obese and diabetics patients. Besides, low-GI meal decreases postprandial blood glucose, triglycerides and total cholesterol, and damage caused to the pancreatic islets [36]. Theoretically, the bulking and viscosity properties of dietary fiber, from whole grain food such as GM, LĐ, and HR, can prevent food intake due to the promotion of satiation and satiety. Fiber-rich foods increase time/efforts of mastication causing a reduction of digestibility and an increase of satiety. Furthermore, GABA, vitamin E, and *γ*-oryzanol in whole grain food can exert hypocholesterolemia in mice fed on HFD [36].

### 3.5. Histopathological Studies of Liver and White Adipose Tissues

**Figure 3:**
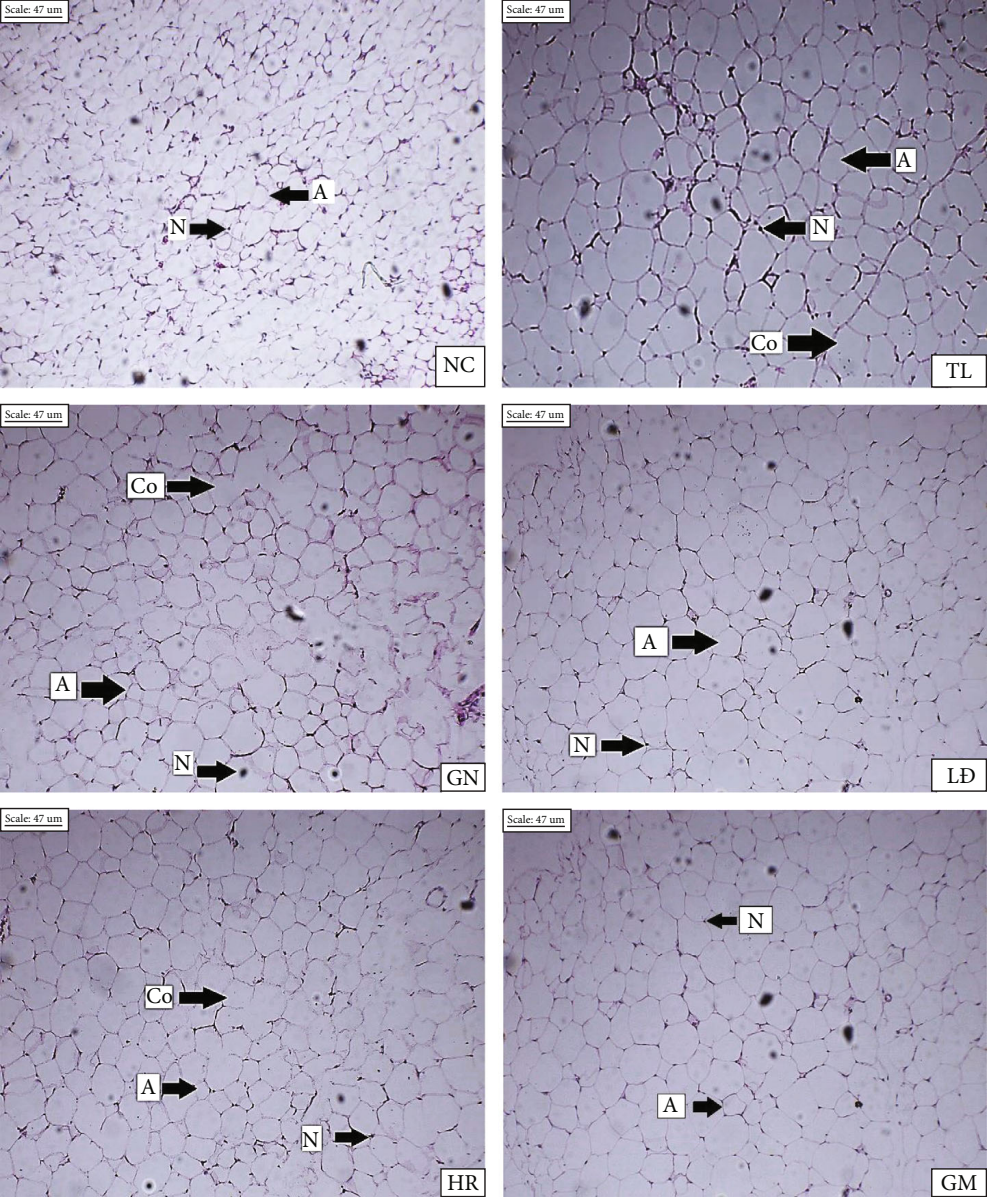
Photomicrographs of white adipose tissue (×100) (A: adipocytes; Co: coalescence; N: nucleus).

Evidently, in this study, the weight of liver and fat between groups had no significant difference (*p* < 0.05, Table 3). White adipose histology of the mice group was shown in Figure 3. Compared to the others, the adipose tissue of untreated HFD mouse (NC) showed the small size of adipocytes, and none of coalescence of adipocytes (Co) (adjacent adipocytes cells fuse together) was seen in this group. All HFD diet groups contained large size of adipocytes. Practically, TL, GN, and HR exhibited the coalescence in their adipose histology. Interestingly, the coalescence was not found in adipose histology of LĐ and GM groups.

Lipid droplet is structured by a monolayer of phospholipids, neutral lipid core, and associated proteins. The development of obesity, fatty liver, impaired insulin sensitivity, and some other diseases is results of insufficient lipid storage (adipose tissue) [38]. Besides, Camastra et al. [39] stated the hypothesis for cyst-like structures (giant crown-like structures) formation “very large adipocytes with breakage of their very thin cytoplasmic rim coalesce forming very large lipid droplets remnants ready to be reabsorbed by macrophages attracted by secreted chemoattractant.” Crown-like structures and/or and cyst-like structures are in subcutaneous fat of obese diabetic patient and frequently found in the adipose tissue of obese individuals (about 30 times more frequent in obese than lean fat). Practically, treatments of LĐ and GM resulted in a reduction of adipocytes coalescence, suggesting the potential role of these rice varieties in restoring the liver damage caused by HFD diet [8].

**Figure 4:**
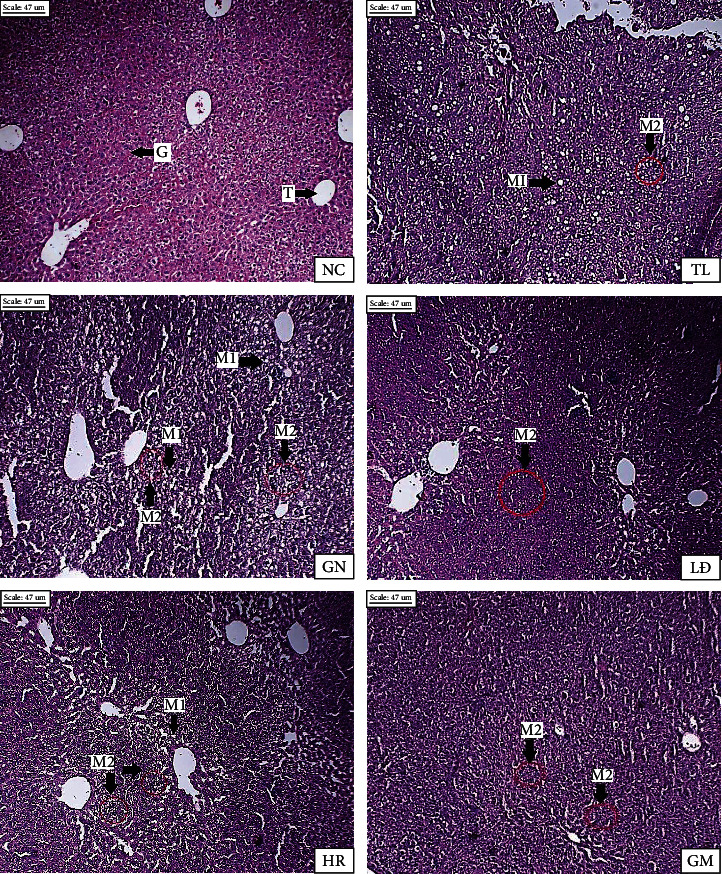
Photomicrographs of white adipose tissue (×100). (T: central vein; G: hepatocytes; M1: macrosteatosis; M2: microsteatosis).

Liver histology of mice groups was shown in Figure 4. Liver histology of untreated HFD mouse (NC) did not contain any evidence of fat droplets. However, high-fat-induced groups showed evidences of micro or macrosteatosis, especially in the case of TL and GN. Actually, in this study, the consumption of HFD diets contributed to obesity leading to various risks of nonalcoholic fatty liver disease in obese individuals and progresses to steatohepatitis, eventually to cirrhosis. However, GM liver histology evidently showed very little signal of microsteatosis, suggesting the potential role of this rice in restoring the liver damage caused by HFD diets [8].

## 4. Conclusions

The results of this study showed that jasmine rice (TL) and golden flower glutinous rice (GN) have a high glycemic index because of their low amylose and crude fiber contents. This is especially dangerous for diabetic patients because of using regularly, the patient's blood sugar will not be controlled. In contrast, germ rice (GM) and brown rice (LĐ), that contain high amylose and fiber contents, are low glycemic index carbohydrate source. In this study, compared to high-GI rices, low-GI rices caused lower triglycerides and LDL/HDL in blood. Low-GI rices also resulted in lower size and weight of white adipose. Furthermore, very little signal of microsteatosis was found in GM and LĐ rices. Therefore, choosing low-GI rices, such as GM and LĐ, to use in daily meals is essential for patients with weight problems, diabetic, or cardiovascular diseases.

## Figures and Tables

**Table 1:** Composition of high-fat diets (HFD)^1^.

Composition (g)	TL	GN	LĐ	HR	GM
Whey protein (Fonterra, New Zealand)	20	20	20	20	20
Lard	30	30	30	30	30
Rice flour	40.5	40.5	40.5	40.5	40.5
Carboxymethyl cellulose (Shandong Yulong, China)	4	4	4	4	4
Vitamin and mineral mix (Doppelherz A-Z Depot, Germany)	5	5	5	5	5
L-Cysteine (Kwang Myung Pharm, Korea)	0.2	0.2	0.2	0.2	0.2
Choline bitartrate (Douglas Laboratories, USA)	0.2	0.2	0.2	0.2	0.2
Methionine (Mekophar, Vietnam)	0.1	0.1	0.1	0.1	0.1
Total	100	100	100	100	100

^1^Note: TL: jasmine rice (Xuan Hong Co. Ltd, Vietnam); GN: golden flower glutinous rice (Xuan Hong Co. Ltd., Vietnam); LĐ: brown rice (Xuan Hong Co. Ltd., Vietnam); HR: red rice (An Gia Khang Co. Ltd., Vietnam); GM: germinated brown rice (Loc Troi Group, Vietnam).

**Table 2:** Chemical composition of rices^1^.

Components (%, w/w)	Jasmine rice (TL)	Golden flower glutinous rice (GN)	Brown rice (LĐ)	Red rice (HR)	Germ rice (GM)
Moisture	12.96 ± 0.15^d^	11.46 ± 0.05^b^	11.86 ± 0.10^c^	12.03 ± 0.12^c^	10.85 ± 0.07^a^
Total carbohydrate	87.15 ± 1.21^b^	90.77 ± 0.97^d^	87.87 ± 1.08^bc^	89.81 ± 0.77^cd^	81.97 ± 1.46^a^
Crude protein	6.22 ± 0.09^a^	6.14 ± 0.13^a^	7.61 ± 0.12^b^	7.60 ± 0.15^b^	8.89 ± 0.10^c^
Crude fat	1.20 ± 0.08^b^	0.88 ± 0.07^a^	1.73 ± 0.03^c^	1.62 ± 0.05^c^	2.03 ± 0.06^d^
Ash	0.23 ± 0.01^a^	0.65 ± 0.06^b^	1.19 ± 0.04^c^	1.08 ± 0.03^d^	1.34 ± 0.02^e^
Crude fiber	0.60 ± 0.02^b^	0.41 ± 0.02^a^	1.14 ± 0.04^d^	0.97 ± 0.02^c^	1.60 ± 0.01^e^
Starch	86.56 ± 1.22^b^	90.36 ± 0.98^c^	86.74 ± 1.11^b^	88.84 ± 0.77^c^	80.37 ± 1.47^a^
Amylose	15.99 ± 0.84^b^	2.86 ± 0.27^a^	21.63 ± 0.91^d^	18.56 ± 0.66^c^	20.68 ± 0.87^d^

^1^Different letters in the same row indicate significantly different means (*p* < 0.05).

**Table 3:** Caloric intake and glycemic index of tested groups^1^.

Group	Glucose	TL	GN	LĐ	HR	GM
Caloric intake	150.0 ± 2.0^f^	110.8 ± 1.1^d^	139.0 ± 2.0^e^	80.6 ± 1.0^b^	102.5 ± 0.9^c^	77.3 ± 1.1^a^
Glycemic index	100.0 ± 0.2^f^	73.9 ± 0.4^d^	92.7 ± 0.2^e^	53.7 ± 0.2^b^	68.4 ± 0.3^c^	51.6 ± 0.2^a^
	Reference food	High	High	Low	Medium	Low

^1^Different letters in the same row indicate significantly different means (*p* < 0.05).

**Table 4:** Total cholesterol (TC), high-density lipoprotein cholesterol (HDL), low-density lipoprotein cholesterol (LDL), triglycerides (TG), weight of liver, and adipose tissues of mice after 5 weeks of feeding HFD^1^.

Group	TL	GN	LĐ	HR	GM
TC (mg/dl)	99.0 ± 10.0^b^	129.8 ± 14.6^e^	101.8 ± 7.8^c^	106.0 ± 8.6^d^	80.0 ± 4.8^a^
TG (mg/dl)	121.2 ± 8.2^c^	132.4 ± 5.6^e^	114.2 ± 8.9^a^	121.4 ± 9.3^d^	116.0 ± 10.3^b^
HDL (mg/dl)	52.8 ± 5.4^b^	48.8 ± 5.2^a^	68.4 ± 9.6^d^	66.4 ± 5.1^c^	73.8 ± 2.2^e^
LDL	48.6 ± 11.5^d^	57.6 ± 5.7^e^	25.4 ± 9.0^b^	33.8 ± 10.8^c^	22.4 ± 4.0^a^
LDL/HDL	0.94 ± 0.31^e^	0.85 ± 0.36^d^	0.39 ± 0.18^b^	0.51 ± 0.18^c^	0.35 ± 0.10^a^
TC/HDL	2.3 ± 0.3^d^	2.7 ± 0.4^e^	1.7 ± 0.2^b^	1.8 ± 0.2^c^	1.5 ± 0.1^a^
Liver (g)	1.29 ± 0.13^a^	1.40 ± 0.33^a^	1.31 ± 0.37^a^	1.27 ± 0.20^a^	1.35 ± 0.23^a^
Adipose (g)	0.90 ± 0.12^a^	1.04 ± 0.38^a^	0.79 ± 0.33^a^	0.88 ± 0.28^a^	0.69 ± 0.27^a^

^1^Different letters in the same row indicate significantly different means (*p* < 0.05).

## Data Availability

(1) The figure data used to support the findings of this study are included within the article. (2) The table original data used to support the findings of this study are included within the article. (3) The [citations] data used to support the findings of this study are included within the article, which have been cited and listed in References.

## Additional Points

*Highlight.* Vietnamese LĐ and GM rice varieties significantly reduced risk of obesity and type 2 diabetes. The higher the amylose and fiber contents were, the lower glycemic index, triglycerides, LDL, and LDL/HDL values were. Physiological value and health benefits were ordered as GN < LT < HR < LĐ < GM.
